# Amine functionalized benzene based hypercrosslinked polymer as an adsorbent for CO_2_/N_2_ adsorption

**DOI:** 10.1038/s41598-023-36434-4

**Published:** 2023-06-06

**Authors:** Mohammad Reza Moradi, Alireza Torkashvand, Hamid Ramezanipour Penchah, Ahad Ghaemi

**Affiliations:** School of Chemical, Petroleum and Gas Engineering, Iran University of Science and Technology, PO Box 16846-13114, Tehran, Iran

**Keywords:** Chemical engineering, Environmental chemistry

## Abstract

In this work, benzene based hypercrosslinked polymer (HCP) as an adsorbent was modified using amine group to enhance CO_2_ uptake capability and selectivity. Based on BET analysis result, the HCP and the modified HCP provide surface area of 806 (m^2^ g^−1^) and micropore volume of 453 (m^2^ g^−1^) and 0.19 (cm^3^ g^−1^) and 0.14 (cm^3^ g^−1^), respectively. The CO_2_ and N_2_ gases adsorption were performed in a laboratory scale reactor at a temperature between 298 and 328 K and pressure up to 9 bar. The experimental data were evaluated using isotherm, kinetic and thermodynamic models to identify the absorbent behavior. The maximum CO_2_ adsorption capacity at 298 K and 9 bar was obtained 301.67 (mg g^−1^) for HCP and 414.41 (mg g^−1^) for amine modified HCP. The CO_2_ adsorption thermodynamic parameters assessment including enthalpy changes, entropy changes, and Gibbs free energy changes at 298 K were resulted − 14.852 (kJ mol^−1^), − 0.024 (kJ mol^−1^ K^−1^), − 7.597 (kJ mol^−1^) for HCP and − 17.498 (kJ mol^−1^), − 0.029(kJ mol^−1^ K^−1^), − 8.9 (kJ mol^−1^) for amine functionalized HCP, respectively. Finally, the selectivity of the samples were calculated at a CO_2_/N_2_ composition of 15:85 (v/v) and 43% enhancement in adsorption selectivity at 298 K was obtained for amine modified HCP.

## Introduction

Excess CO_2_ emissions from fossil fuel burning cause severe global climate and environmental challenges, drawing attention to carbon capture and storage (CCS) technologies throughout the world^1,2^. Over several decades, the CO_2_ capture and storage using liquid amines has been developed as a applicable methods and is now employed in a variety of industrial applications^3,4^. However, this method has some drawbacks, including high recovery energy demanding, corrosion problems, and liquid amine losses during absorption process^5,6^. To tackle the mentioned issues, researchers have been focused deeply on developing solid sorbents for CO_2_ capture propose due to their lower recovery energy requirements, high adsorption capacity, selective behavior in gas separation, and stable performance in the adsorption–desorption cycles^7^. Porous organic polymers (POPs) are functional materials having low skeletal density, large specific surface area, and stable physical and chemical properties which have extensive applications in gas storage, chemical catalysis, separation, drug delivery, and any other potential field^8^. (POPs) are classified into covalent organic frameworks (COFs)^9,10^, conjugated microporous polymers (CMPs)^11,12^, covalent thiazine frameworks (CTFs)^13,14^, metal–organic frameworks (MOFs)^15,16^, polymers of intrinsic microporosity (PIM)^17,18^, hypercrosslinked polymers (HCPs)^19,20^, and so on^21^. HCPs are a class of intriguing platform due to their high specific surface areas, chemical stability, and good thermal stability, and high affordability^22^. Generally, HCPs are generated through excessive crosslinking of aromatic monomers via Friedel–Crafts alkylation reaction, resulting in a stretched polymer which can be remained porous when the solvent is removed^23,24^.

There is currently significant interest in the use of Hypercrosslinked polymer for CO_2_ capture and storage and gas separation applications^24^. For example, Hassan, et al.^25^ synthesized Triptycene based and nitrogen rich hypercrosslinked polymers (TNHCP-1), which resulted a CO_2_ adsorption capacity of 98 mg g^−1^. Hui Gao et al.^26^, prepared pitch-based HCP sample and investigated CO_2_ adsorption which yielded the CO_2_ uptake capacity of 17.74 wt% at 1.0 bar and 273 K. According to the findings of the similar researches on the CO_2_ uptake by polymeric adsorbents, it can be concluded that improving the adsorbents surface’s chemistry increases CO_2_ adsorption capacity and selectivity through improving intermolecular interactions between the CO_2_ molecules and the adsorbent surface’s functional groups^7,27–33^. Therefore, the incorporation of the heteroatoms such as N, O, S, etc. improve the HCP sample’s surface potential heterogeneity which causes increasing in CO_2_ uptake capacity and selectivity^34,35^. Adding amine groups to a solid adsorbent is an effective approach to improve selectivity because it increases the affinity for CO_2_ adsorption through chemisorption mechanism^33^. Such functional groups incorporation on POPs precursors is a time-consuming task because, in the most cases, the functional groups existence on the POPs precursors could not endure the polymerization conditions, or due to the functional groups’ incompatibility with the polymerization reaction the polymerization was unsuccessful^36^. A post-synthetic modification is one of the most effective ways to solve this issue^37^. The benefits of adding amine groups to solid adsorbents have attracted increasing attention to the development of amine/porous material composites^38^. Chemical modification and physical impregnation are typically the two main methods used to functionalize solid adsorbents. Although chemical modification is an easier method than physical impregnation, the adsorbent functionalized by chemical modification at higher temperatures has better chemical stability than the physical impregnation method ^29^. For example Krishnan et al.^39^ provided an amine-modified micro porous HCP adsorbent (PCP-1) with a CO_2_ uptake capacity of 103.8 mg g^−1^ at 273 K and 1 bar. Najafi et al. prepared a microporous polymer that has been impregnated with ethylene diamine (B-Cl-1). The result shows the CO_2_ adsorption capacity of 39.15 mg g^−1^ at 273 K and 1 bar^29^.

The present study introduces a novel adsorbent for CO_2_ capture from CO_2_/N_2_ mixtures, specifically targeting industrial flue gas streams. A hyper-crosslinked polymer adsorbent was developed, and its CO_2_ capture capability was significantly enhanced through amine grafting. Characterization techniques, including FTIR, XPS, EDS, and BET analysis, were employed to investigate the morphological surface properties of the adsorbent. The investigation of the CO_2_ adsorption mechanism using the FTIR spectroscopy technique shed light on the surface properties and role of amine incorporation on the CO_2_ adsorption. Moreover, this research provides insightful information about the field of CO_2_ adsorption process by developing isotherm and kinetic models. The modeling approaches investigate the adsorption mechanisms and dynamic behavior of CO_2_ adsorption. Moreover, these models have practical implications for industrial process design applications, allowing for optimization and scaling of CO_2_ capture systems. Additionally, the thermodynamic feasibility of the adsorption process for both types of samples was explored, providing valuable insights into the energy requirements and efficiency of the CO_2_ desorption step. Utilizing the Ideal Adsorbed Solution Theory (IAST), the research predicted the adsorption performance of the adsorbent for the typical composition of CO_2_/N_2_ such as 15:85 (v/v) found in industrial flue stacks. The findings of this research contribute to the development of more efficient and sustainable CO_2_ capture technologies, with potential applications in large-scale industrial settings.

## Experimental procedure

### Materials

Benzene, anhydrous iron (III) chloride, nitric acid (65%), sulfuric acid (98%), formic acid, sodium hydroxide, 1,2-dichloro ethane (DCE), formaldehyde dimethyl acetal (FDA), ethanol, and iron Nano powder (average, 25 nm) were supplied from Merck company. During the adsorbent synthesis and modification procedure, distillated water and ethanol were used for washing. All of the mentioned materials were consumed without further purification.

### HCP synthesis

**Figure 1:**
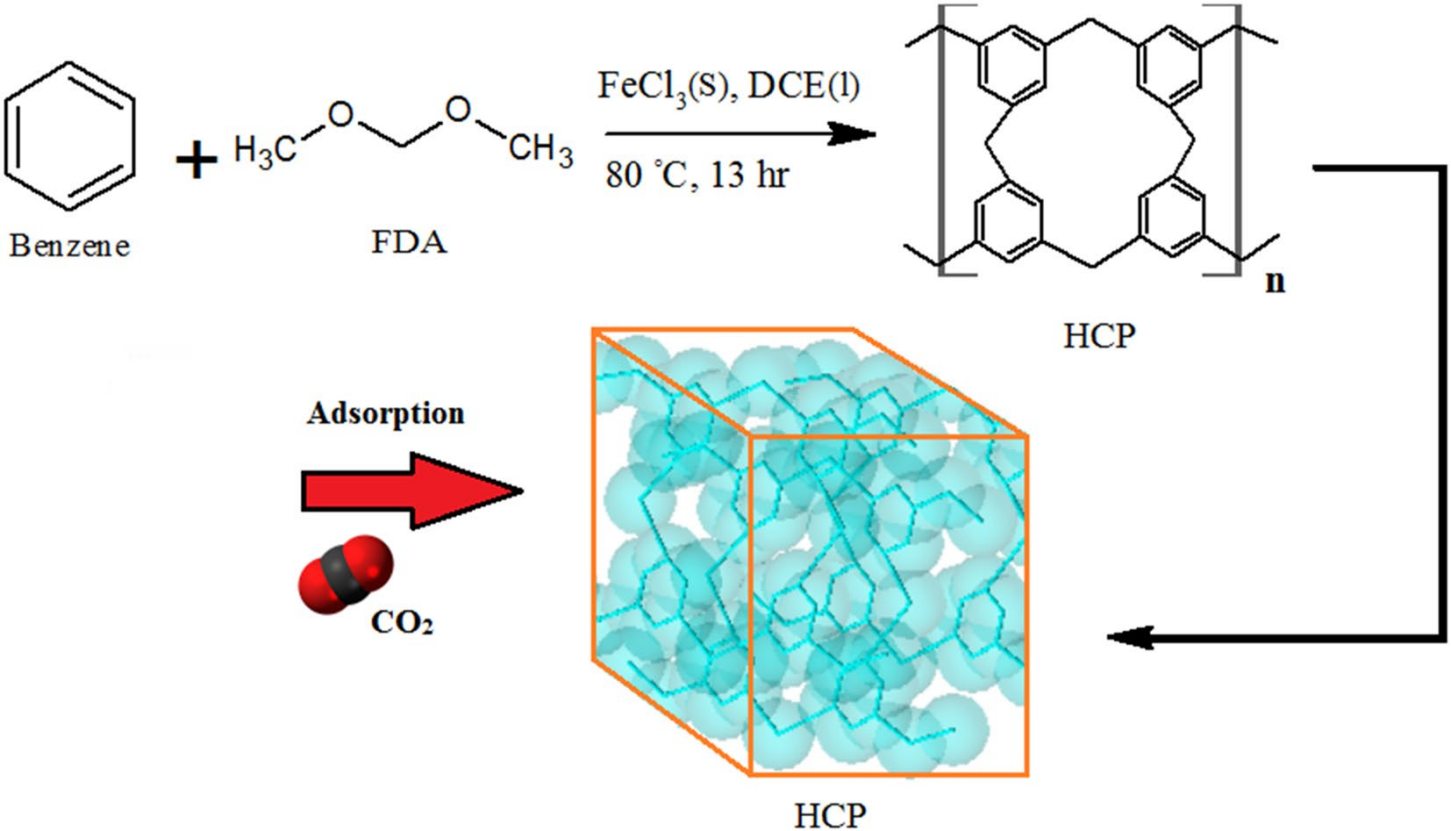
Benzene based HCP synthesis procedure.

The benzene based hypercrosslinked polymeric adsorbent (HCP) was synthesized through the “knitting” method which was reported by Li et al.^23^. To achieve the highest specific surface area and the highest capability of gas adsorption by HCP adsorbent, the synthesis parameters such as synthesis time and cross linker to benzene ratio were considered in the optimal condition that was reported by Ramezanipour et al.^40^. In a general method, benzene (0.02 mol), 1,2-dichloro ethane (30 ml), and FDA (0.06 mol) were entered to a three-Neck flask and the flask contents were blended at room temperature in presence of the nitrogen atmosphere for 15 min. Then the iron (III) chloride (0.06 mol) was added to the mixture and the resulting mixture was stirred at 40 °C for 3 h. After 3 h, the mixture temperature was gained to 80 °C, and the flask content was stirred in a nitrogen atmosphere and reflux condition for 13 h. Finally, the flask content was cooled down to room temperature and the resulting polymeric network was filtrated and purified with deionized water and ethanol using the soxhlet extractor apparatus for 15 h. The purified HCP was dried in an oven and vacuum condition at 150 °C for 12 h, which yielded brown powder. The HCP adsorbent synthesis procedure is illustrated in Fig. 1.

### Adsorbent modification through HCP amination

The HCP adsorbent amination was carried out to increase nitrogen sites in the HCP adsorbent structure. It should be considered that through a hyper crosslinking reaction with the FDA to benzene molar ratio of 3, the three carbons of the six carbons which exist in the benzene ring will be incorporated into neighborhood benzene molecules by cross linker molecule. So, the three available carbon atoms in each benzene ring have the potential to be nitrated and make nitro benzene molecules in the adsorbent network^41^. In a typical procedure, the HCP adsorbent modification was carried out through primary amine (–NH_2_) synthesis on benzene aromatic ring in two steps. In the first step, nitro group (–NO_2_) incorporation into the benzene aromatic ring was take place through electrophilic substitution of benzene molecules by nitronium ions (NO_2_^+^)^42^. The nitronium ion formation and aromatic nitration mechanism are represented in Eq. (1), and Eq. (2)^42,43^. To perform nitration the benzene molecules which yielded nitrated HCP adsorbent (Nitro-HCP), a mixture of HCP (5 gr), nitric acid 14.3 M (8.8 ml), sulfuric acid 2 M (5 ml), and deionized water (50 ml) were charged in a round bottom flask. Then, the flask contents were blended at 55 °C for 10 h in reflux condition and then the flask contents were filtrated and purified with deionized water several times to remove the excess acids which stuck in adsorbent pores. The purified nitro functionalized HCP was dried in an oven at 120 °C for 10 h which yielded the light orange powder.

**Figure 2:**
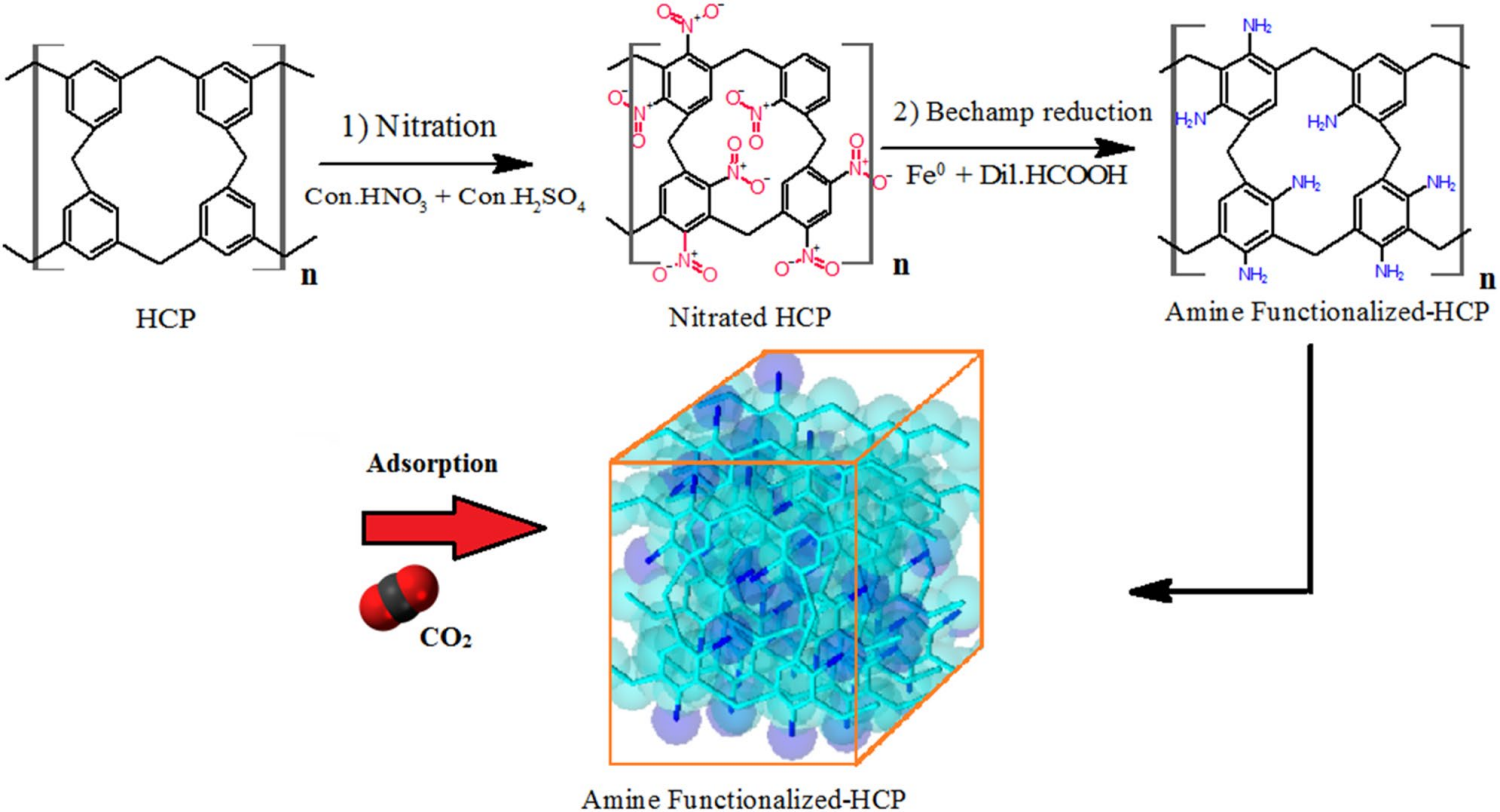
Amine functionalization procedure of the benzene based HCP.

In the second step, the nitro group (–NO_2_) reduction to an amine group (–NH_2_) takes place through the “Bechamp reduction” reaction which reported by Popat et al.^44^. The nitro group reduction (Bechamp reaction) mechanism is represented in Eq. (3)^44^. To synthesis the aminated HCP adsorbent, a mixture of Nitro-HCP powder (5 gr), iron nanopowder (16 gr), formic acid 0.01 M (0.2 ml), and deionized water (300 ml) were charged in a round bottom flask. The mixture was blended using a 400 W ultrasound device with 20 kHz frequency (400 W, Fanavari Iranian Pajouhesh Nasir Company, Iran) at a pH of 5.1 and temperature of 100 °C for 2 h in reflux conditions. After 2 h, the flask content was filtrated and washed with an excess amount of NaOH solution (0.1 M) and deionized water several times until neutral pH and finally the filtrated network was dried in a vacuum oven at 140 °C for 12 h. The HCP amination procedure is illustrated in Fig. 2.

1 $${\text{HNO}}_{3} { } + { }2{\text{ H}}_{2} {\text{SO}}_{4} { } \to {\text{H}}_{3} {\text{O}}^{ + } + {\text{ NO}}_{2}^{ + } + { }2{\text{ HSO}}_{4}^{ - }$$

2 $${\text{ArH}} + {\text{NO}}_{2}^{ + } \to {\text{Ar}} - {\text{NO}}_{2} + {\text{H}}^{ + }$$

3 $$4{\text{Ar}} - {\text{NO}}_{2} + 4{\text{H}}_{2} {\text{O }} + { }9{\text{Fe}}^{0} \to 4{\text{ArNH}}_{2} + 3{\text{Fe}}_{3} {\text{O}}_{4}$$

### Adsorbent characterization

In order to characterize the elemental composition of HCP and amine modified HCP adsorbents, the energy dispersive X-ray spectroscopy (EDS) analysis was carried out by Philips- × 130 instrument, also the X-ray photoelectron spectroscopy (XPS) analysis was utilized by an Al Kα source (XPS spectrometer Kratos AXIs Supra) instrument. To investigate the adsorbents morphology and pores size characterization, the nitrogen adsorption and desorption analysis was done at 77 K by ASAP 2020 M analyzer and FTIR analysis was performed by PerkinElmer FTIR spectrometer instrument.

### Adsorption setup

**Figure 3:**
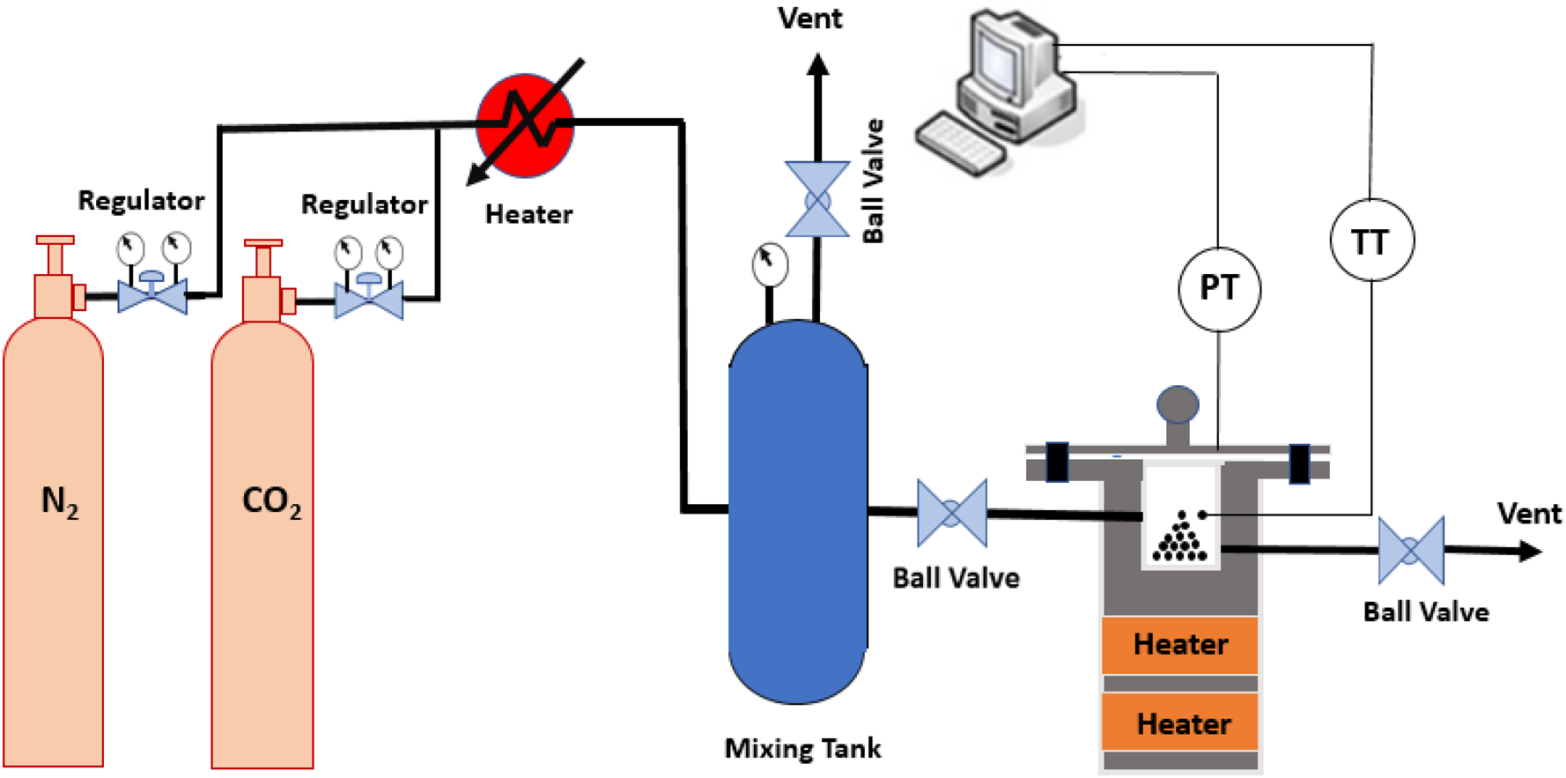
Experimental CO_2_ adsorption setup.

According to Fig. 3, CO_2_ or N_2_ gases with a high purity of 99.99% exits from the gas storage cylinder and the gas is heated trough passing inside an electrical heater. Then the gas enters into the mixing tank, at the mixing tank the gases temperature and the pressure become uniform, and then the gas is transferred to the reactor where the gas meets the adsorbent. The pressure and temperature sensors installed on the reactor measure the gas pressure and temperature and provide the data to the controller. The reactor temperature is maintained at the set point temperature thanks to the controller’s adjustment of heating duty, and the temperature and the pressure data are recorded in the computer device every second. Equation (4) represent the adsorption capacity calculation.

4 $$q = \left( {\frac{{V \times {\text{Mw}}}}{R \times W}} \right) \times \left( {\left[ {\frac{P}{{Z \times {\text{T}}}}} \right]_{i} - \left[ {\frac{P}{{Z \times {\text{T}}}}} \right]_{f} } \right)$$

5 $$Z = 1 + \frac{B \times P}{{R \times T}}$$

which i and f represent the initial and the final condition. P, V, R, T, M_w_, W, and Z are the pressure, reactor volume, global gas constant, temperature, gas molecular weight, the mass of adsorbent, and compressibility factor, respectively. The B parameter refers to the virial second coefficient calculated using the Tsonopoulos correlation^45^ where represented in Eqs. (6–8). The operational conditions, which considered as effective parameters on CO_2_/N_2_ adsorption process, were summarized in Table 1.

6 $$B = \frac{{R \times T_{c} }}{{P_{c} }} \left( {F^{\left( 0 \right)} (T_{r} } \right) + {\upomega }F^{\left( 1 \right)} (T_{r} ))$$

7 $$F^{\left( 0 \right)} (T_{r} ) = 0.1445 - \frac{0.330}{{T_{r} }} - \frac{0.1385}{{T_{r}^{2} }} - \frac{0.0121}{{T_{r}^{3} }} - \frac{0.000607}{{T_{r}^{8} }}$$

8 $$F^{\left( 1 \right)} (T_{r} ) = 0.0637 + \frac{0.331}{{T_{r}^{2} }} - \frac{0.423}{{T_{r}^{3} }} - \frac{0.008}{{T_{r}^{8} }}$$

**Table 1:** The operational condition for the process parameters.

Parameters	Unit	Lower limit	Higher limit
Adsorption time	Sec	0	3600
Temperature	K	298	328
Pressure	Bar	1	9

### Ideal adsorbed solution theory (IAST)

The applicable approach for binary mixture computations is the ideal adsorbed solution theory (IAST) which was introduced by Myers and Prausnitz^46^. According to this theory, the adsorbed phase is considered as ideal phase without interaction in binary mixture systems. Based on this theory, for a specific adsorbent at a fixed temperature, only pure components adsorption isotherms are sufficient to calculate the molar fraction of component j (x_j_) and the total amount of adsorbed components (n_t_) in adsorbed phase^47^. In this theory, the spreading pressure (P^*^) is defined as a hypothetical pressure of adsorbed phase components on the adsorbent surface. For a two-component gaseous system (a, b), the IAST approach starts by linking the spreading pressure (P_j_^*^) to system composition in both gas phase (y_j_) and adsorbed phase (x_j_). The calculation procedure is summarized in Eq. (9).

9 $$P y_{a} = P_{a}^{*} x_{a}$$

10 $$x_{b} = \frac{{P_{a}^{*} - P}}{{P_{a}^{*} - P_{b}^{*} }}$$

11 $$\mathop \smallint \limits_{0}^{{P_{a}^{*} }} \frac{{n_{a} \left( P \right)}}{P} dP = \mathop \smallint \limits_{0}^{{P_{b}^{*} }} \frac{{n_{b} \left( P \right)}}{P} dP$$

12 $$\frac{1}{{n_{t} }} = \frac{{x_{a} }}{{n_{a} \left( {P_{a}^{*} } \right)}} + \frac{{1 - x_{a} }}{{n_{b} \left( {P_{b}^{*} } \right)}}$$

where n(P), x, y, P, P^*^, and n_t_ refer to the adsorption isotherm model, molar fraction in adsorbed phase, molar fraction in the gas phase, total pressure, spreading pressure, and total quantity of adsorbed phase, respectively. Solving the set of mentioned equations for $P_{a}^{*}$ and $P_{b}^{*}$ will result in all of the information about system composition^48^.

## Results and discussion

### Adsorbents characterization

**Figure 4:**
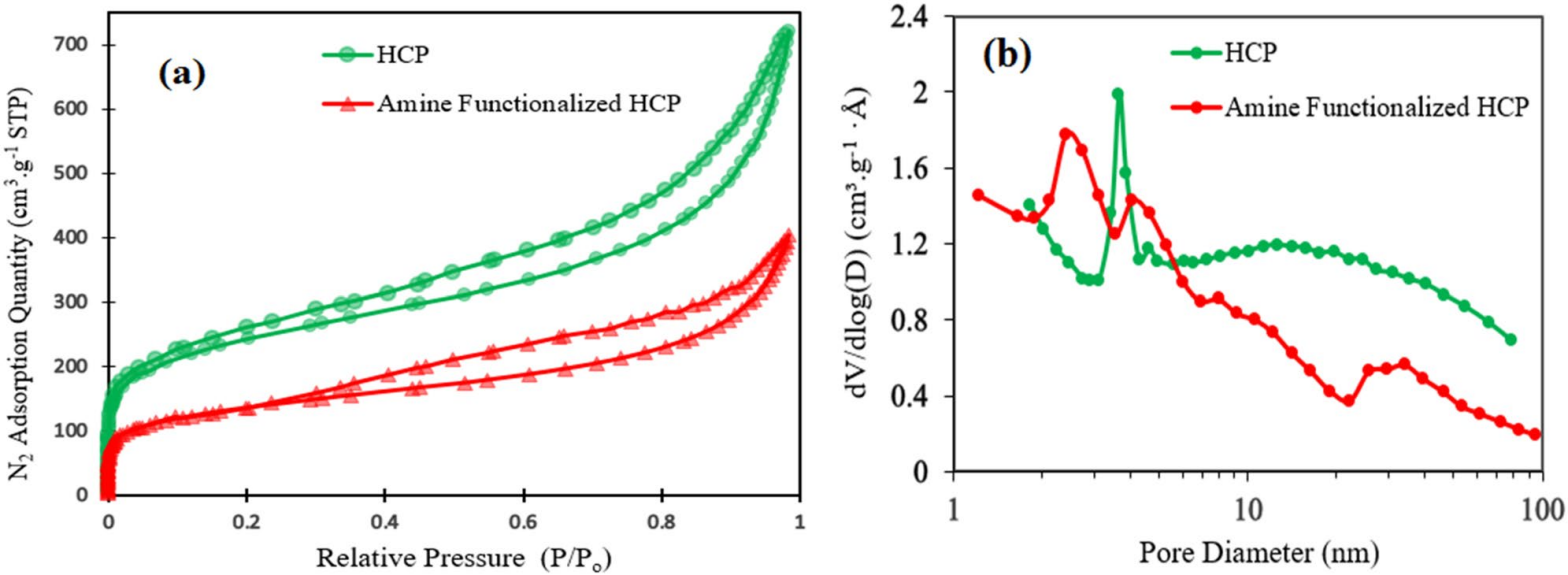
(**a**) Nitrogen adsorption–desorption isotherm curves for HCP and amine functionalized HCP adsorbents, (**b**) pore size distribution curves (calculated based on BJH method).

**Figure 5:**
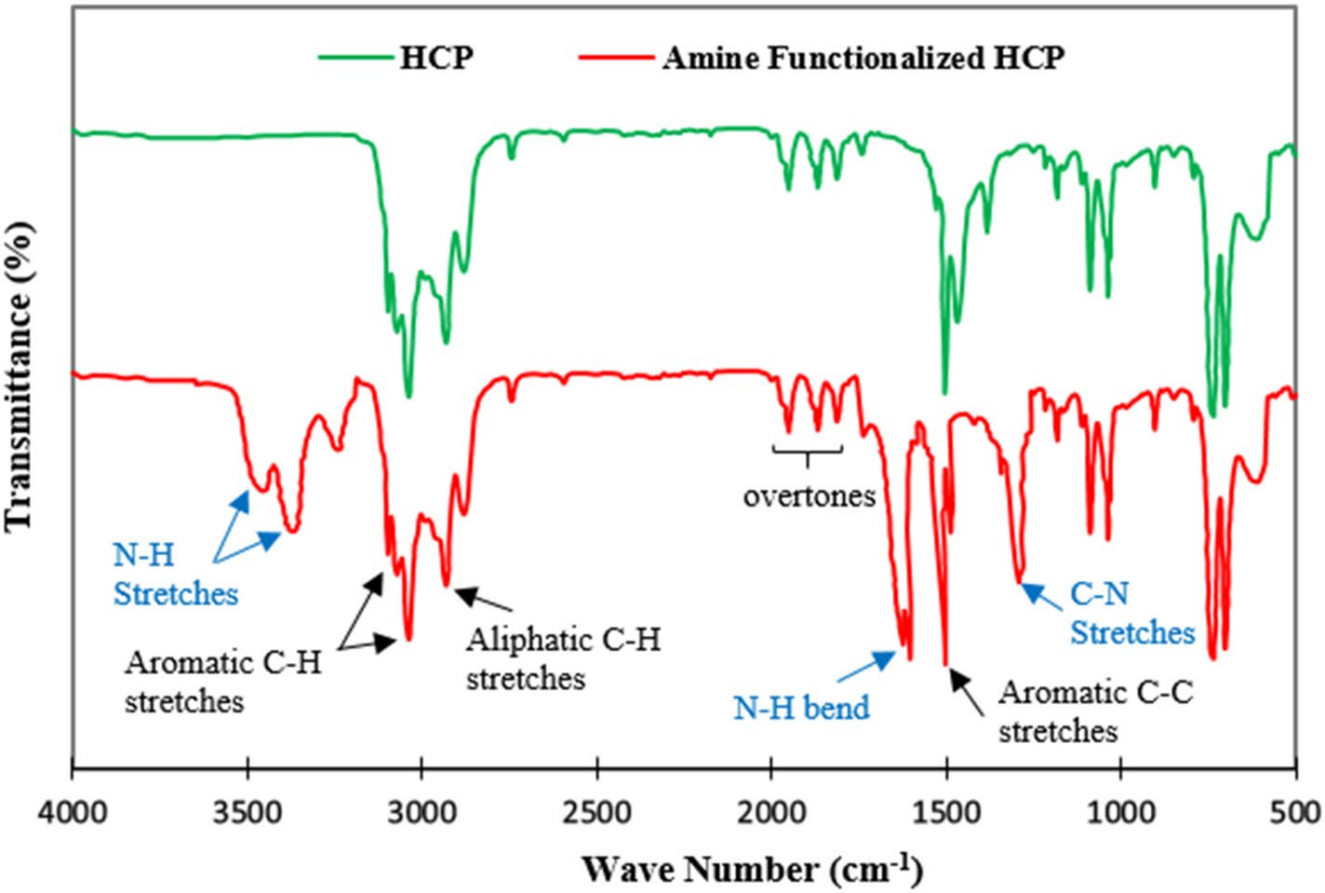
FTIR spectra of HCP and amine functionalized HCP adsorbents.

**Figure 6:**
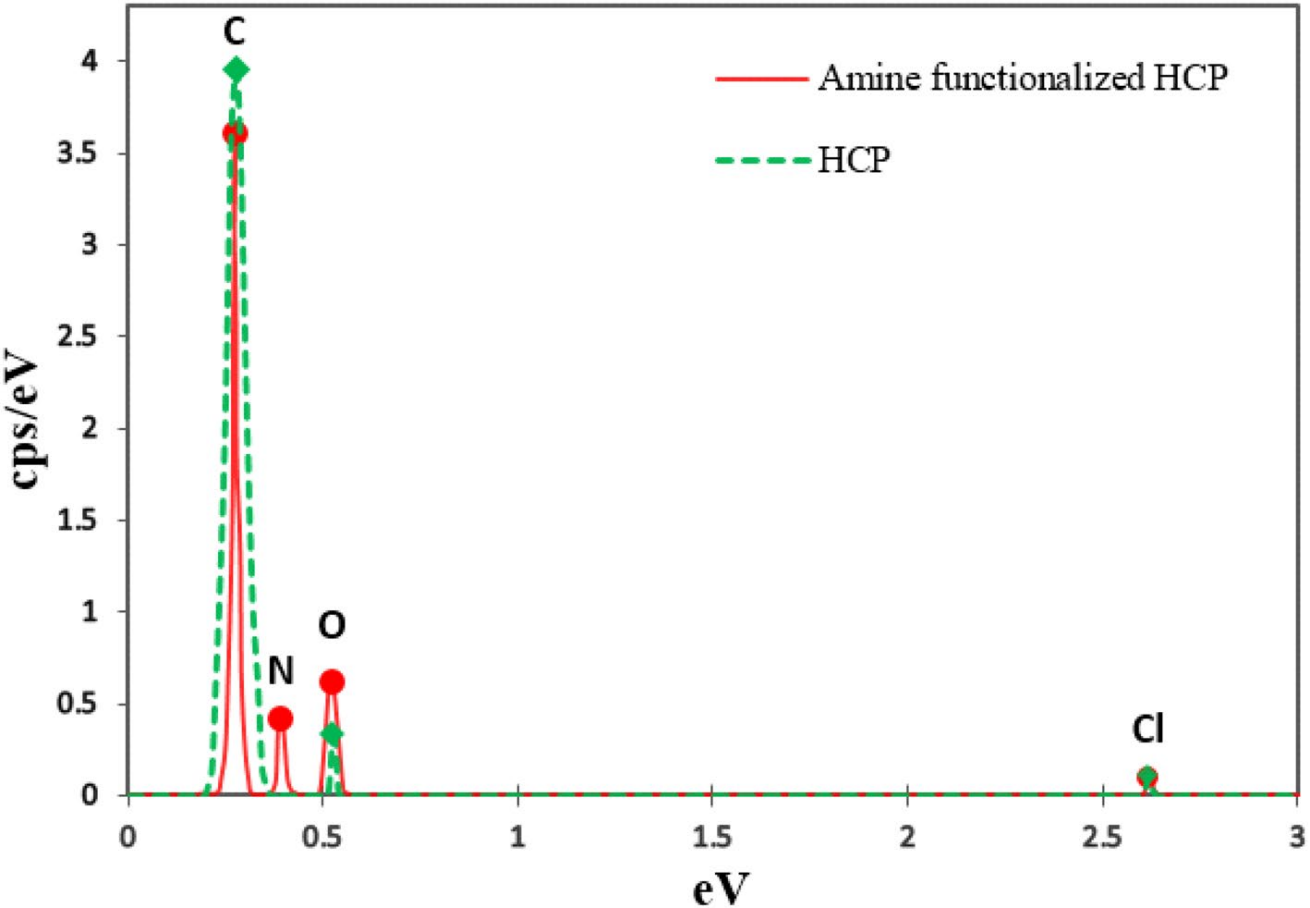
EDX elemental analysis of HCP and amine functionalized HCP adsorbents.

**Figure 7:**
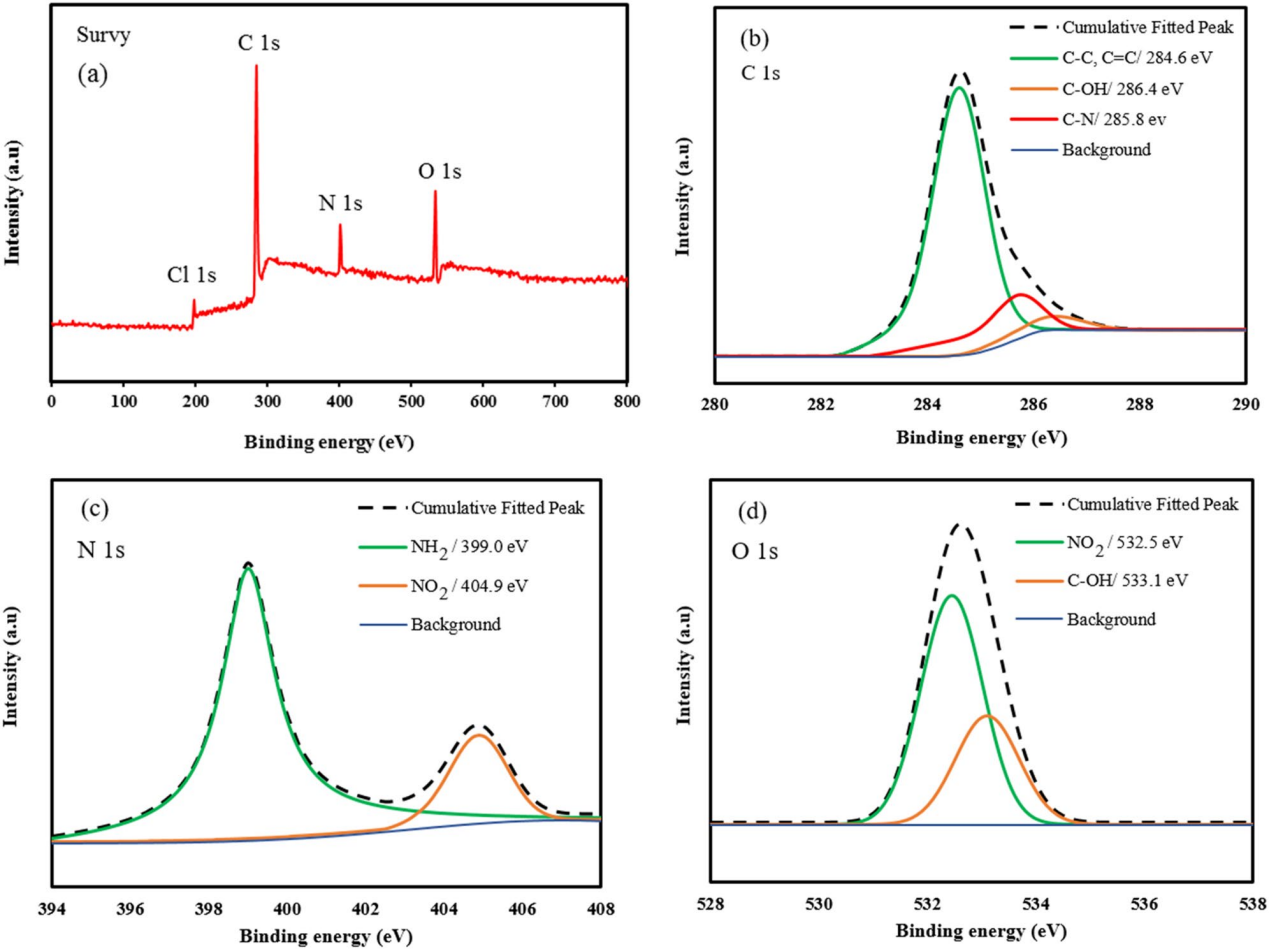
XPS analysis results of the amine functionalized HCP sample (**a**) survey scan spectrum of the sample (**b**) High resolution C 1s spectrum, (**c**) High resolution N 1s spectrum, (**d**) High resolution O 1s spectrum.

The porosities of both types of adsorbents were measured by the Nitrogen adsorption–desorption isotherms at 77.3 K which are shown in Fig. 4a. According to Fig. 4a, rapid adsorption of Nitrogen by both types of adsorbents at relative pressure lower than 0.05 refer to the existence of micropores in the structure of the adsorbent, while the hysteresis loop presence at a higher relative pressure (0.2 < P/P_0_ < 0.8) proves the mesoporous character. The hysteresis loop at a high relative pressure (P/P_0_ > 0.8) refers to the existence of inter particle cavities and macropores in polymer structure^49^. The detailed porosity properties of the adsorbents are summarized in Table 2. The BET surface area of the adsorbents are in descending order of HCP (806 m^2^/g) > amine functionalized HCP (453 m^2^/g). The surface area reduction in amine modified HCP may be related to partial filling of the volume of the pores by the amine group^50^. The pore size distribution curves of adsorbents are shown in Fig. 4b. According to Fig. 4b, for HCP adsorbent, a pore diameter peak is observed at 3.59 nm and for amine modified HCP two peaks are observed at 2.53 nm and 4.05 nm which has good accordance with hysteresis loop presence in nitrogen adsorption isotherms. The result of the pore size distribution curve of modified HCP refers to some changes in pore structure after functionalization. Accordingly, the incorporation of the amine group may separate mesopores into multiple micropores which makes the resulting HCP more favorable for CO_2_ adsorption application^51^. The FTIR spectra of the adsorbents are shown in Fig. 5. In the spectrum of the amine modified HCP, the peaks at 3442 cm^−1^ and 3360 cm^−1^ are related to primary amine N–H stretches, the peak at 1619 cm^−1^ is related to primary amine N–H bending, and the peak at 1281 cm^−1^ is related to C–N stretches in amines functional group^52^. The result of the spectrum proves the successful incorporation of the amine group into the HCP structure. The result of EDX analysis is shown in Fig. 6 for both types of the adsorbents. According to EDX elemental analysis results, the HCP sample ingredients include 94.74% carbon, 3.91% oxygen, and 1.31% chlorine elements which are related to Friedel–Crafts reaction. After amine functionalization, the EDX result shows that the modified HCP sample contains 86.48% carbon, 7.42% oxygen, 1.17% chlorine, and 4.93% nitrogen which proves the successfully incorporation of the amine group to the HCP network structure. The enhancement of the oxygen atom percentage in modified HCP may be related to the unreacted nitro (–NO_2_) group in the polymer structure. To better assessment of the amine modified HCP sample’s characteristics, the XPS analysis was applied in the range of 0 to 800 eV which is illustrated in Fig. 7. According to the Fig. 7a, four peaks can be observed at 198.5 eV, 285.5 eV, 400.4 eV, and 533.7 eV which are correspond to Cl 1 s (1.11%), C 1s (86.66%), N 1s (5.17%), and O 1s (7.06%), respectively. The chlorine element (1.11%) can be found as ionic form (Cl^−^) which was remained from the Lewis-acid catalyst used in the Friedel–Crafts reaction^53^. Based on the high resolution spectra of the C 1s element which is shown in the Fig. 7b, in can be concluded that the C 1s peak deconvoluted to three peaks that are related to C–C/C=C bonds (284.6 eV), C–OH bond (286.4 eV), and C–N bond (285.8 eV). Deconvoluted peaks of the N 1 s spectra which is shown in Fig. 7c, are attributed to the amine group N (–NH_2_ peak at 399.0 eV) and the nitro group N (–NO_2_ peak at 404.9 eV). Base on the findings, about 3.48% of the total nitrogen element can be existed as the amine group in the amine functionalized HCP sample skeleton and 1.69% of the total nitrogen can be existed as the nitro group. According to the Fig. 7d, the O 1s spectra consists of two distinct peaks that are related to the N–O bond in the nitro group (–NO_2_ peak at 532.5 eV) and the C–OH bond (533.1 eV)^54–57^.

**Table 2:** Porous properties of the HCP and amine functionalized HCP adsorbents.

Adsorbent	S_BET_^a^ (m^2^/g)	MPV^b^ (cm^3^/g)	MPA^c^ (m^2^/g)	PV^d^ (cm^3^/g)	APW^e^ (nm)
HCP	806	0.19	192	1.09	5.41
Amine functionalized HCP	453	0.14	129	0.61	5.39

^a^specific surface area (calculated based on BET model at 77.3 K).

^b^micropore volume.

^c^micro surface area.

^d^total pore volume.

^e^average pore width (calculated based on BET and 4 V/A equation).

### Adsorption isotherm modeling

**Figure 8:**
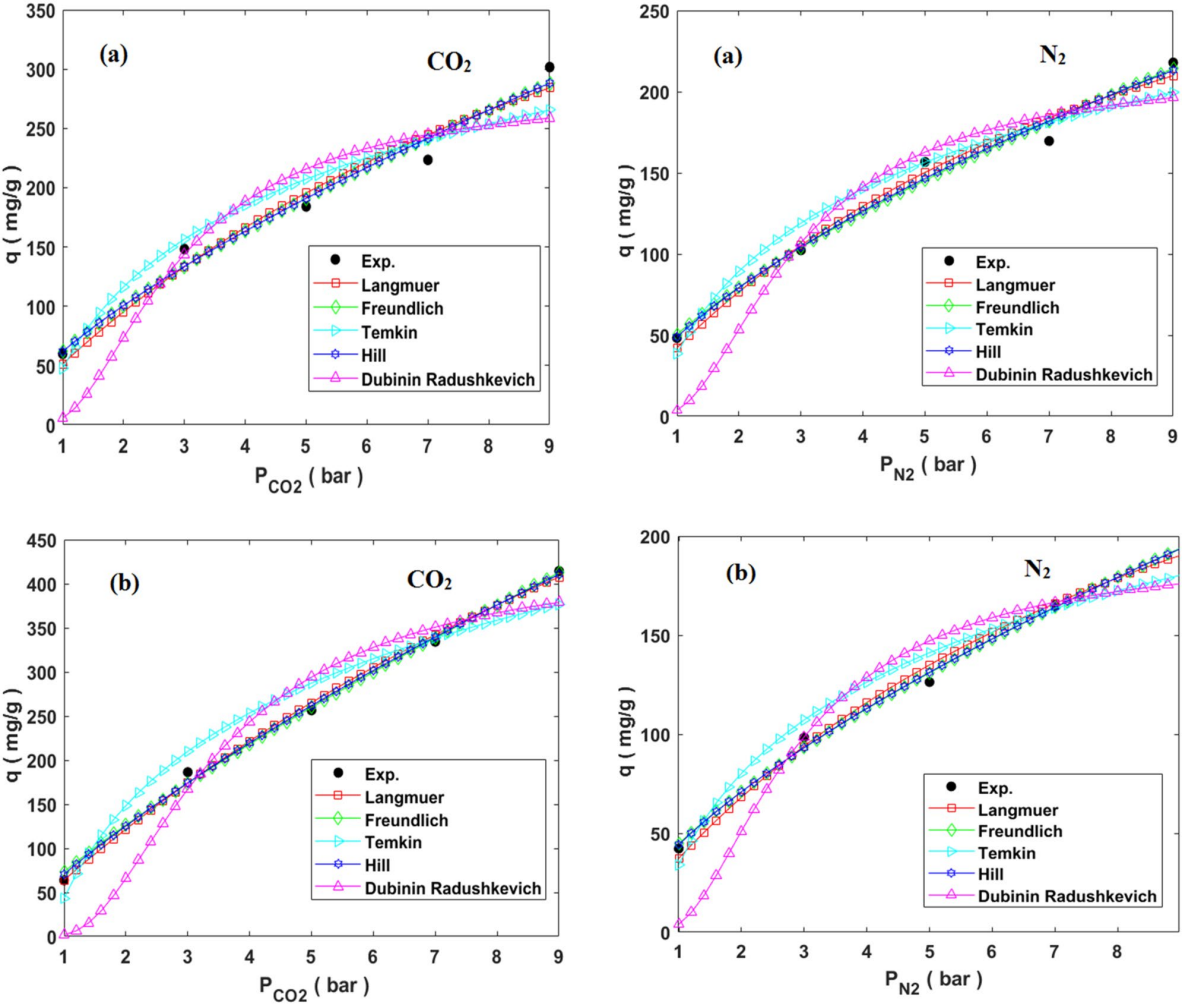
comparison of isotherms models and experimental values of CO_2_/N_2_ adsorption at the temperature of 298 K by (**a**) HCP, (**b**) amine modified HCP.

To investigate the CO_2_/N_2_ adsorption behavior of HCP and N-HCP adsorbents, the equilibrium isotherm modeling was carried out. Isotherm modeling was used to determine the adsorbent's affinity and surface characteristics, which is important for adsorption system design. The following Dubinin–Radushkevich, Temkin, Freundlich, Langmuir, and Hill isotherm models which are represented in Eqs. (13–17), respectively, were used to fit the experimental CO_2_ and N_2_ adsorption capacity by both types of the adsorbents. The mentioned models were plotted in Fig. 8 at a constant temperature of 298 K and pressure interval between 1 and 9 bar. In addition, the models parameters and correlation coefficients (R^2^) at temperatures of 298 K, 308 K, and 318 K were reported in Table 3.

13 $${\text{Langmuir: }}q_{e} = \frac{{q_{m} K_{l} P_{e} }}{{1 + K_{l} P_{e} }}$$

14 $${\text{Freundlich: }}q_{e} = k_{F} P_{e}^{{{\raise0.7ex\hbox{$1$} \!\mathord{\left/ {\vphantom {1 {n_{F} }}}\right.\kern-0pt} \!\lower0.7ex\hbox{${n_{F} }$}}}}$$

15 $${\text{Temkin: }}q_{e} = B Ln A_{T} + B Ln P_{e} ,\;B = \left( {\frac{RT}{{b_{T} }}} \right)$$

16 $${\text{Hill: }}q_{e} = \frac{{q_{m} P_{e}^{{n_{H} }} }}{{K_{H} + P_{e}^{{n_{H} }} }}$$

17 $${\text{Dubinin}} - {\text{Radushkevich: }}q_{e} = q_{m} {\text{exp}}\left( { - \lambda \omega ^{2} } \right)$$

where q_e_ and q_m_ are the equilibrium amount and maximum amount of CO_2_/N_2_ adsorption capacity (mg g^−1^), p_e_ is the pressure at equilibrium state (bar), K_F_ (mg g^−1^ bar^1/n^) and n_F_ are the Freundlich model constants, K_l_ is the Langmuir model constant, K_H_ (bar^1/n^) and n_H_ are the Hill model constants. The term $\lambda$ in the (D–R) model is model constant (mol^2^ J^−1^) and the term $\omega$ refers to Polanyi potential (J mol^−1^), A_T_ (L mol^−1^) is the constant of Temkin model and the term B refers to first virial coefficient ($B = \left( {\frac{RT}{{b_{T} }}} \right), b_{T} = \left( {J.mol^{ - 1} } \right) )$^58^. According to the finding of Table 3, Freundlich model constant K_F_ which relates to the affinity of adsorbate-adsorbent, is reduced by the increasing temperature that refers to the physisorption mechanism predominance over chemisorption mechanism for the adsorption of CO_2_/N_2_ by both types of the adsorbents, in addition adsorption capacity reduction by temperature increment proves the exothermic behavior of adsorption process. Furthermore, Freundlich constant n range between 1–2 represents the favorability of CO_2_/N_2_ adsorption^59^. Additionally, the $\omega$ value below 8 kj/mol that calculated based on the Dubinin-Radushkevich model suggests the physically adsorption of gases on the adsorbents surface. Based on average R^2^ values of isotherm models, the Freundlich model has best accuracy than another, which implies that the adsorbent's surface are heterogeneous and the adsorption process occurs as multilayer on the surface^60^.

**Table 3:** Isotherm model parameters of CO_2_/N_2_ uptake by HCP and amine modified HCP.

Model	Parameters	HCP						N-HCP					
		CO_2_			N_2_			CO_2_			N_2_		
		298 K	308 K	318 K	298 K	308 K	318 K	298 K	308 K	318 K	298 K	308 K	318 K
Langmuir	$q_{m}$	655.3	418.94	276.87	416.53	323.64	190.95	1240.6	921.76	536.08	390.3	251.64	104.99
	$K_{l}$	0.085	0.1375	0.1871	0.113	0.129	0.22	0.0543	0.0622	0.1151	0.1057	0.1498	0.4401
	R^2^	0.982	0.996	0.993	0.99	0.997	0.995	0.997	0.998	0.991	0.996	0.996	0.985
Freundlich	$K_{F}$	61.89	59.99	51.95	50.414	44.563	41.27	73.27	62.69	64.66	44.66	39.7	36.31
	$n_{F}$	1.428	1.596	1.777	1.519	1.589	1.905	1.271	1.31	1.502	1.497	1.671	2.502
	R^2^	0.987	0.996	0.996	0.991	0.999	0.994	0.998	0.999	0.985	0.998	0.992	0.994
Temkin	$\frac{RT}{{b_{T} }}$	99.16	80.86	57.23	73.225	29.238	40.586	151.64	121.38	99.38	66.5	48.161	23.157
	$A_{T}$	1.616	1.761	2.14	1.695	1.833	2.4	1.33	1.392	1.551	1.67	1.988	4.319
	R^2^	0.963	0.989	0.987	0.975	0.982	0.988	0.971	0.977	0.983	0.98	0.982	0.991
Hill	$q_{m}$	614.8	649.4	971.68	144.3	263.59	304.98	323.65	366.4	434.11	392.89	1148.4	314.13
	$K_{H}$	99.02	10.7	18.191	28.501	58.84	6.935	44.85	58.9	7.654	87.8	28.541	7.817
	$n_{H}$	0.716	0.819	0.64	0.727	0.658	0.739	0.853	0.809	1.157	0.69	0.653	0.498
	R^2^	0.987	0.993	0.995	0.991	0.995	0.992	0.996	0.999	0.982	0.998	0.996	0.994
D–R	$q_{m}$	283.2	216.49	159.55	215.65	160.7	117.04	430.09	349.93	285.12	192.38	131.97	79.183
	$\lambda$	1.334	0.684	0.458	1.38	0.672	0.415	1.863	1.708	1.276	1.314	0.559	0.274
	$\omega$	0.612	0.855	1.045	0.602	0.863	1.098	0.518	0.541	0.626	0.617	0.946	1.35
	R^2^	0.933	0.939	0.923	0.959	0.92	0.918	0.971	0.972	0.977	0.956	0.91	0.926

### Adsorption kinetic modeling

**Figure 9:**
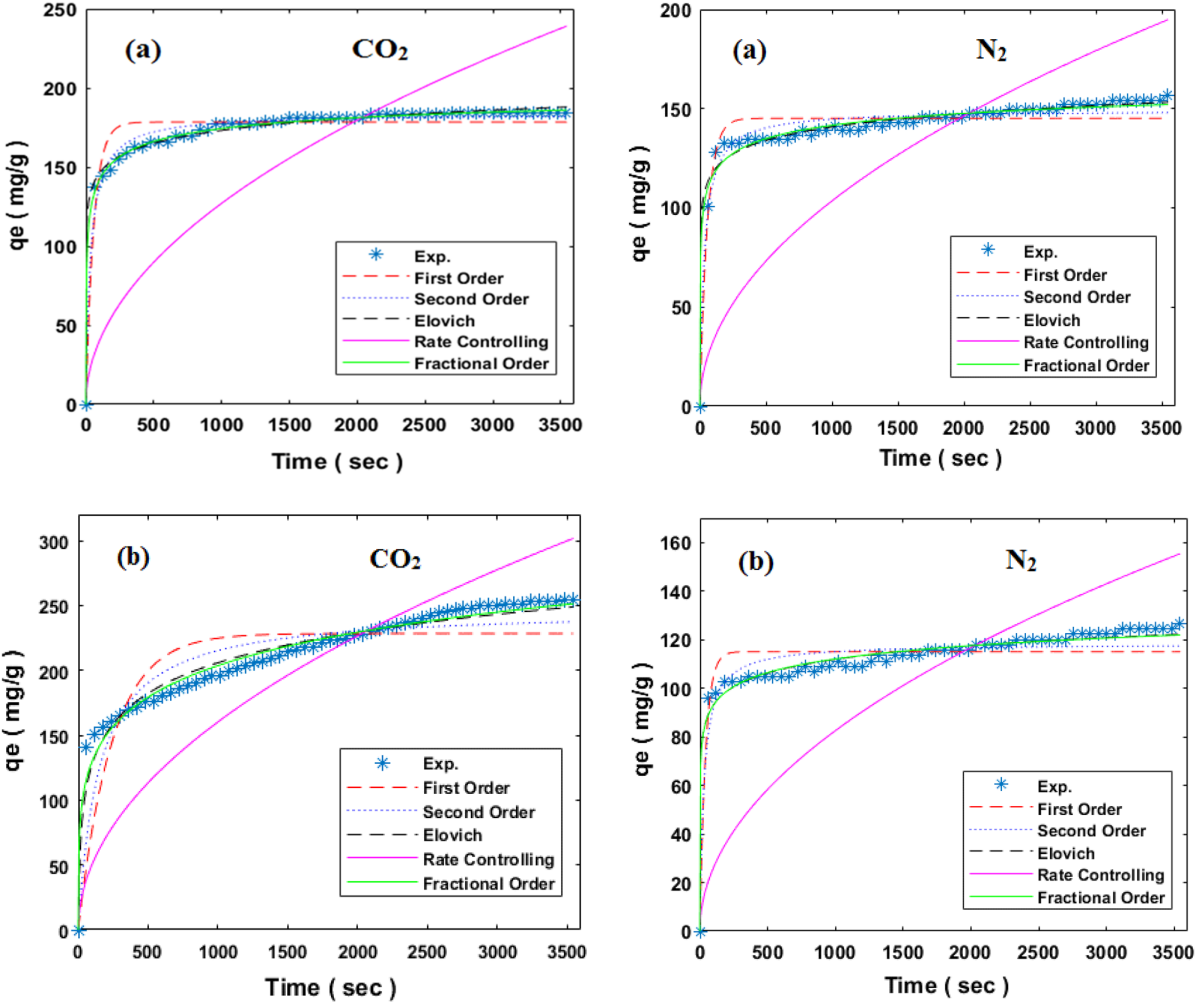
Comparison of kinetic models and experimental values of CO_2_/N_2_ adsorption at the temperature of 298 K and pressure of 5 bar by (**a**) HCP, (**b**) amine modified HCP.

The gaseous molecules adsorption processes on porous materials surfaces are influenced by surface heterogeneity, interconnected porosities, and the microporous or mesoporous structure of the adsorbent. Physical and chemical properties of adsorbent surface have a key role in determining the adsorption mechanism. To study the adsorption kinetic, some theoretical models including first order, second order, fractional order, rate controlling, and Elovich models, which are represented in Eqs. (18–22), respectively, were fitted with experimental data and plotted in Fig. 9. In addition, the model parameters and correlation coefficients of CO_2_/N_2_ adsorption at temperatures of 298 K, 308 K, 318 K and pressure of 5 bar were reported in Table 4.

18 $${\text{First order: }}q_{t} = q_{e} \left( {1 - e^{{k_{1} t}} } \right)$$

19 $${\text{Second order: }}q_{t} = \left( {q_{e}^{2} k_{2} t} \right)/\left( { 1 + q_{e} k_{2} t} \right)$$

20 $${\text{Fractional order: }}q_{t} = q_{e} - \left[ {\frac{{\left( {n - 1} \right)}}{m} k_{n} t^{m} + q_{e}^{{\left( {1 - n} \right)}} } \right]^{{\left( {1/\left( {1 - n} \right)} \right)}}$$

21 $${\text{Rate controlling: }}q_{t} = k_{c} t^{0.5}$$

22 $${\text{Elovich: }}q_{t} = \beta \ln \left( {\alpha \beta } \right) + \beta {\text{ln}}\left( t \right)$$

where q_t_, k_1_, k_2_, and k_n_ refer to adsorption capacity, first order model rate constant, second order model rate constant, and fractional order model rate constant, respectively. The terms m, n, $\alpha$, and $\beta$ are the kinetic model parameters^61^. The first-order model is based on the assumption that the rate of change of solute uptake with time is directly proportional to difference in saturation concentration and the amount of solid uptake with time, which shows physical adsorption process. A decrease in R^2^ of latter model as shown in Table 4 indicates increasing role of chemical adsorption on the adsorption process^62^. The Rate Controlling Model was extensively utilized for the analysis of mass transfer mechanisms, which established intraparticle diffusion as the exclusive determining factor in regulating the rate of the process. From Table 4 is shown that R^2^ of this model has increased after amine modification implying that diffusion is the rate controlling process. This can be attributed to decrease of pore sizes after amine modification which is also shown in N_2_ adsorption/desorption section^63^. According to the finding of Table 4 and correlation coefficient (R^2^) values of the kinetic models, the fractional order can be chosen as the best model for describing the relationship between CO_2_/N_2_ adsorption capacity and reaction time. The fractional order kinetic model offers a more encompassing and precise depiction of adsorption phenomena that deviate from integer order kinetics. It incorporates considerations of surface heterogeneity, multilayer adsorption, and the interactions between adsorbate molecules, all of which contribute to the intricate nature of the adsorption process^64^

**Table 4:** Kinetic model parameters of CO_2_/N_2_ adsorption at the pressure of 5 bar by benzene based HCP and amine modified HCP.

Model	Parameters	HCP						N-HCP					
		CO_2_			N_2_			CO_2_			N_2_		
		298 K	308 K	318 K	298 K	308 K	318 K	298 K	308 K	318 K	298 K	308 K	318 K
First order	$q_{e}$	178.44	148.39	123.53	145.13	99.175	76.066	223.38	190.75	170.8	115.04	79.28	55.62
	$k_{1}$	0.0171	0.0137	0.0105	0.0177	0.0158	0.0151	0.0042	0.0036	0.0035	0.0241	0.0153	0.0086
	R^2^	0.945	0.936	0.93	0.946	0.899	0.847	0.84	0.838	0.858	0.905	0.85	0.805
Second order	$q_{e}$	183.8	153.64	128.68	149.31	103.02	79.746	247.5	207.5	186.05	118.35	83.06	60.026
	$k_{2}$	0.0002	0.0002	0.0001	0.0002	0.0002	0.0002	3E-05	3E-05	3E-05	0.0003	0.0002	0.0002
	R^2^	0.986	0.984	0.983	0.974	0.95	0.92	0.921	0.914	0.927	0.947	0.93	0.905
Fractional order	$q_{e}$	208.67	178.24	148.44	187.42	162.15	122.38	467.15	414.33	389.52	195.4	144.69	108.27
	$k_{n}$	0.0007	0.0002	0.0003	4E−05	1E−05	4E−05	0.004	0.0005	7E−05	4E−05	2E−05	2E−05
	m	0.375	0.4452	0.462	0.359	0.2754	0.3107	0.2616	0.2892	0.3058	0.1968	0.2684	0.34
	n	1.9822	2.2733	2.1214	2.6057	2.7115	2.5609	1.3085	1.6475	1.9597	2.4583	2.6671	2.5632
	R^2^	0.998	0.998	0.997	0.989	0.988	0.979	0.989	0.985	0.989	0.988	0.982	0.975
Rate controlling	$k_{c}$	4.017	3.339	2.774	3.274	2.248	1.732	5.068	4.206	3.754	2.611	1.805	1.258
	R^2^	0.678	0.714	0.7508	0.704	0.784	0.835	0.931	0.94	0.941	0.741	0.833	0.911
Elovich	$\alpha$	232.37	29.65	4.882	175.75	8.001	1.741	0.013	0.011	0.011	114.73	1.841	0.109
	$\beta$	11.689	11.246	10.843	9.804	8.572	7.667	33.98	29.37	26.75	8.14	7.921	7.667
	R^2^	0.98	0.98	0.98	0.95	0.963	0.9513	0.969	0.96	0.971	0.949	0.951	0.951

### Adsorption thermodynamic analysis

**Figure 10:**
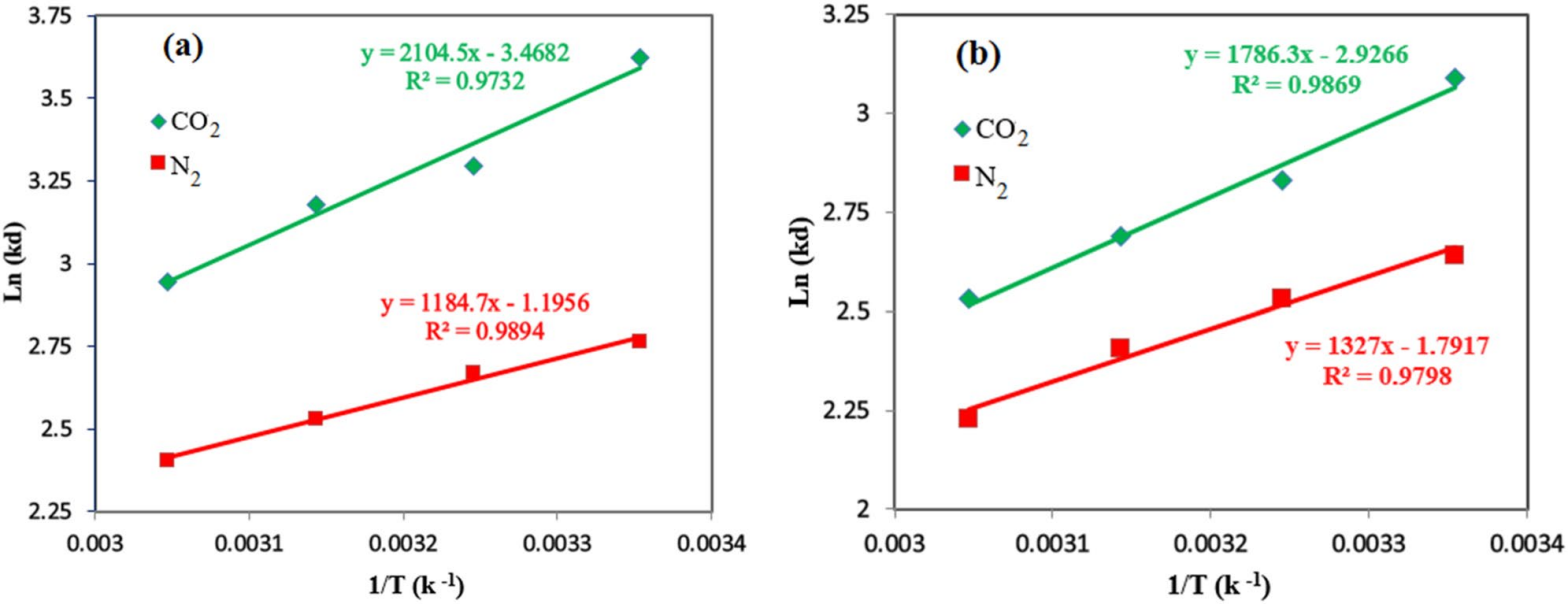
The van’t hoff plots of CO_2_/N_2_ adsorption at pressure of 5 bar by (**a**) Amine functionalized HCP, (**b**) HCP.

Adsorption process thermodynamic analysis was investigated by calculating thermodynamic parameters including Gibbs free energy changes (ΔG), entropy changes (ΔS), and enthalpy changes (ΔH) at temperature 298–328 K and pressure of 5 bar using the following equations:

23 $$K_{d} = \Delta P_{ads} \times \frac{V}{w}$$

24 $$\ln \left( {K_{d} } \right) = \frac{{{\Delta S}^{0} }}{R} - \frac{{{\Delta H}^{0} }}{RT}$$

25 $${\Delta G}^{0} = {\Delta H}^{0} - T{\Delta S}^{0}$$

where $\Delta P_{ads}$, V, W, and R refer to the reactor vessel's initial and final pressure difference, reactor volume, adsorbent weight, and global gas constant (8.314 J mol^−1^ K^−1^), respectively^40^. By plotting the values of ln (K_d_) versus the 1/T values, the Van’t Hoff plots are plotted and shown in Fig. 10. The slope of the Van’t Hoff plot is enthalpy (${\Delta H}^{0}$) and the intercept of the plot is entropy (${\Delta S}^{0}$) values of adsorption, the ${\Delta G}^{0}$ of adsorption can be calculated using Eq. (25). Table 5 depicts the results of the CO_2_/N_2_ adsorption thermodynamic parameters by both type of adsorbents. According to the findings, the negative values of the adsorption enthalpy refer to the exothermic adsorption process. Moreover, the enthalpy of the CO_2_ adsorption by amine modified HCP sample (− 17.498 kJ/mol) shows more negative value than the HCP sample (− 14.852 kJ/mol). By considering a higher heat releases during adsorption process when the aminated HCP used as adsorbent, it can be resulted in that the CO_2_ uptake favorability can be enhanced via improving the HCP surface’s heterogeneity^65^. The (${\Delta S}^{0}$) values of the adsorption process provide significant insights about the randomized or organized relationship between adsorbate molecules and adsorbent surface. It can be more random by positive values of the adsorption entropy (${\Delta S}^{0} > 0$) or less random by negative values of the adsorption entropy (${\Delta S}^{0} < 0$). By considering the negative values of the entropy for all systems, it can be concluded that the gas–solid interface are less random. The negative values of ${\Delta G}^{0}$ for all systems indicate that the adsorption processes are thermodynamically feasible and proceed spontaneously^58^.

**Table 5:** Thermodynamic parameters value of CO_2_/N_2_ adsorption.

Adsorbent	Adsorbate	${\Delta H}^{0}$(kJ/mol)	${\Delta S}^{0}$(kJ/mol K)	${\Delta G}^{0}$(kJ/mol)			
				298 K	308 K	318 K	328 K
Amine functionalized HCP	CO_2_	− 17.498	− 0.029	− 8.900	− 8.612	− 8.324	− 8.03518
	N_2_	− 9.850	− 0.010	− 6.886	− 6.787	− 6.687	− 6.58808
HCP	CO_2_	− 14.852	− 0.024	− 7.597	− 7.354	− 7.111	− 6.86721
	N_2_	− 11.033	− 0.015	− 6.592	− 6.443	− 6.294	− 6.14483

**Figure 11:**
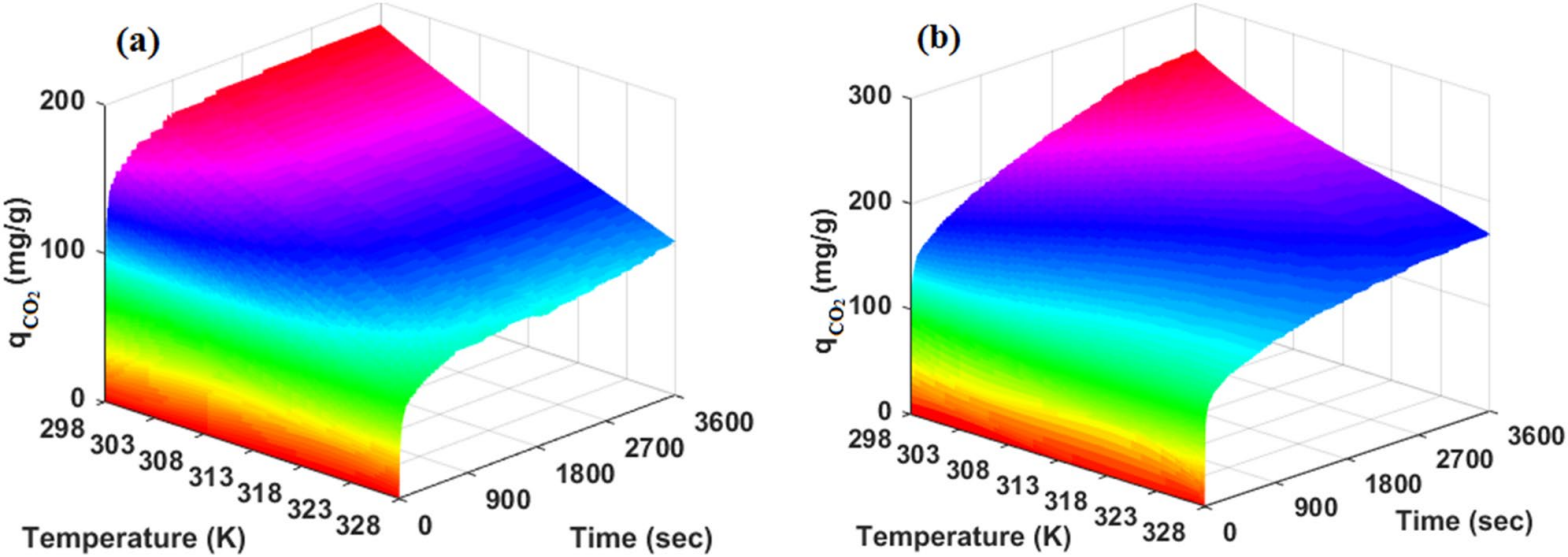
effect of temperature and adsorption time on CO_2_ adsorption performance at pressure of 5 bar, by (**a**) HCP, (**b**) amine functionalized HCP.

To investigate the CO_2_ adsorption performance in both types of adsorbents, the CO_2_ uptake quantities were plotted versus adsorption time and temperature at 5 bar. The results are illustrated in Fig. 11. As can be seen, temperature increasing from 298 to 328 K, decreased CO_2_ uptake capacity and the highest adsorption capacity was observed at 298 K and 3600 Sec in both types of adsorbents. The CO_2_ uptake decrement can be related to the predominance of the CO_2_ molecule physisorption on the adsorbents surface and weak Van der Waals interaction between adsorbent surface and adsorbate molecule. Based on Fig. 11 result, CO_2_ adsorption occurred more quickly on the HCP surface than modified HCP at the same time. It can be concluded that the incorporation of amine groups makes a change in HCP’s surface heterogeneity and improve quadrupole-dipole interaction between CO_2_ and adsorbent surface which causes increasing CO_2_ molecules tendency to adsorb on the surface and improves the mass transfer rate into adsorbent pores.

### Adsorption mechanism

**Figure 12:**
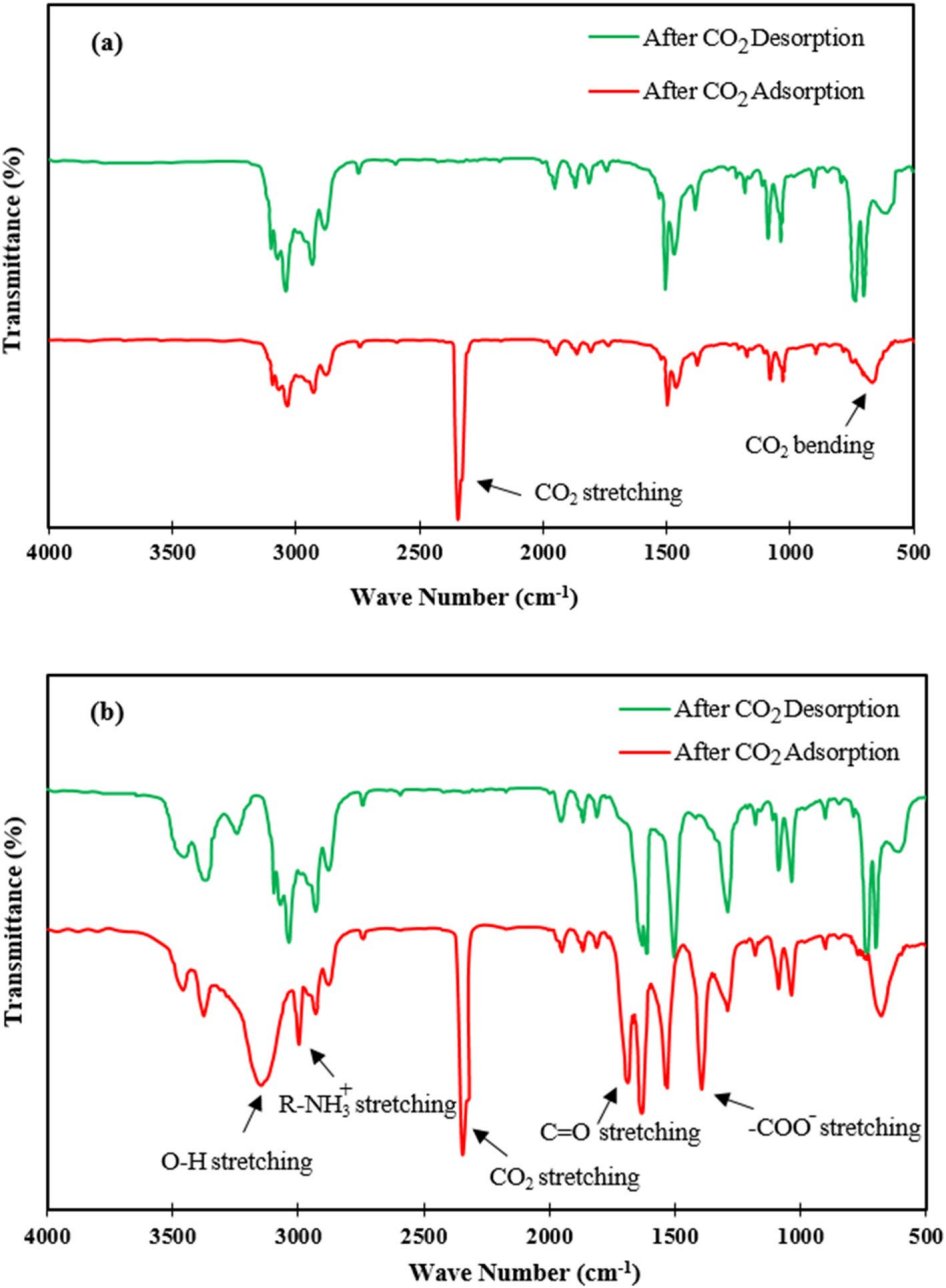
FTIR spectra of the (**a**) HCP sample, (**b**) amine functionalized HCP sample after CO_2_ adsorption/desorption process.

To investigate the CO_2_ adsorption mechanism and the role of amine group in chemically adsorption of the CO_2_ molecules, the FTIR spectra of the both HCP samples were prepared after adsorption/desorption process. According to the FTIR spectra of the HCP sample (Fig. 12a), a sharp peak around 2345 cm^−1^ that is related to the stretching vibration of the CO_2_ molecule, can be observed after the CO_2_ adsorption process. Therefore, it can be concluded that the CO_2_ molecules can be adsorbed by the HCP sample through physisorption mechanism. In contrast, the FTIR spectra of the amine functionalized HCP sample (Fig. 12b) represent the simultaneously CO_2_ adsorption via chemisorption and physisorption mechanisms. In the Fig. 12-b, the sharp peak observed at 2349 cm^−1^ correspond to the CO_2_ stretching vibration which proves the physically adsorption mechanism, meanwhile some new bands which were observed after CO_2_ adsorption refer to chemically adsorption of the CO_2_ molecules by the modified HCP. In the Fig. 12b, the bands observed around 2997 cm^−1^ and 1626 cm^−1^ are related to ammonium formation specially ${\text{R}} - {\text{NH}}_{3}^{ + }$ stretching vibration, and ${\text{R}} - {\text{NH}}_{2}^{ + }$ stretching vibration, respectively. The band presence at 1686 cm^−1^ is related to the C=O bond stretching vibration which proves carbamic acid formation, and also the observed bands around 1532 cm^−1^ and 1686 cm^−1^ correspond to the asymmetric and symmetric stretching vibration of COO^−^, which are attributed to the formation of the carbamate ions^66–68^.

**Figure 13:**
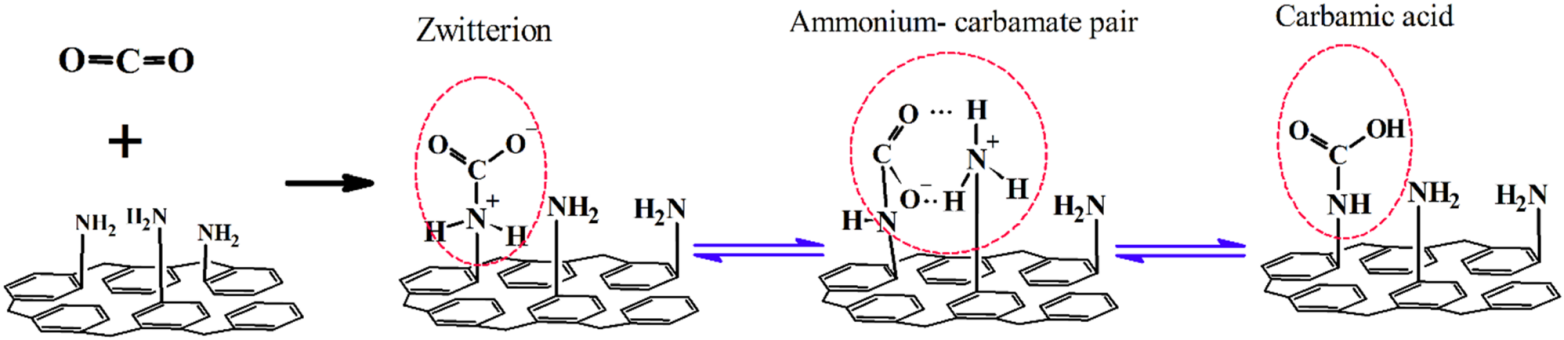
CO_2_ chemisorption mechanism by amine functionalized HCP sample.

Generally, the CO_2_ molecules uptake by amine moieties can be taken place via two step reaction: first the primary amine adsorbed the CO_2_ molecules by zwitterion intermediate (${\text{R}} - {\text{NH}}_{2}^{ + } \cdots {\text{ COO}}^{ - }$) formation. Next, the zwitterion intermediate deprotonation with the neighboring amine moieties result in ammonium-carbamate ion pairs ((${\text{R}} - {\text{NH}}_{3}^{ + } \cdots {\text{ COO}}^{ - } - {\text{NH}} - {\text{R}}$)) formation, also carbamic acid (${\text{R}} - {\text{NH}} - {\text{ COOH}}$) species can be formed through intermolecular proto transfer^68^. Based on the FTIR analysis findings, the FTIR spectra of the amine functionalized HCP sample confirm the mentioned species formation after CO_2_ adsorption process. General procedure of the CO_2_ molecule uptake by chemisorption mechanism is illustrated graphically in Fig. 13.

To study CO_2_ desorption process efficiency, the process was conducted at a temperature of 410 K in a vacuum oven for 8 h. The FTIR spectra of the both HCP samples after CO_2_ desorption process (Fig. 12) confirm the completely desorption of the CO_2_ molecules from the both HCP adsorbents surface.

### CO_2_/N_2_ adsorption selectivity

Basically, the adsorption selectivity of the CO_2_ over N_2_ can be attributed to several molecular properties. Firstly, CO_2_ exhibits a higher quadrupole moment around 4.3 $\times$ 10^−26^ ${\text{esu}}^{ - 1} {\text{cm}}^{ - 1}$ compared to the N_2_ (1.52 × 10^−26^ ${\text{esu}}^{ - 1} {\text{cm}}^{ - 1}$), which results in a stronger van der Waals force between CO_2_ molecules and the adsorbent surface. This stronger interaction allows CO_2_ to adhere more effectively to porous polymer’s surfaces, enhancing its adsorption capacity. Furthermore, CO_2_ is more polarizable than N_2_, with a polarizability value of 29.1 × 10^−25^ cm^3^ for CO_2_ and 17.4 × 10^−25^ cm^3^ for N_2_. This allows CO_2_ molecules to undergo greater distortion in the presence of an electric field, facilitating their adsorption onto the adsorbent material. Moreover, by considering the kinetic diameter values of the N_2_ (0.36 nm) and CO_2_ (0.33 nm), it can be concluded that molecular sieving techniques may not have significant effect on their separation^69,70^. As a result, a combination of the mentioned effects including the CO_2_ molecule’s higher quadrupole moment, and higher polarizability contribute to its enhanced adsorption compared to N_2_. These molecular properties enable CO_2_ to form stronger interactions with the adsorbent with a more heterogeneous surface, resulting in a higher selectivity for CO_2_ over N_2_ during the adsorption process^71^.

**Figure 14:**
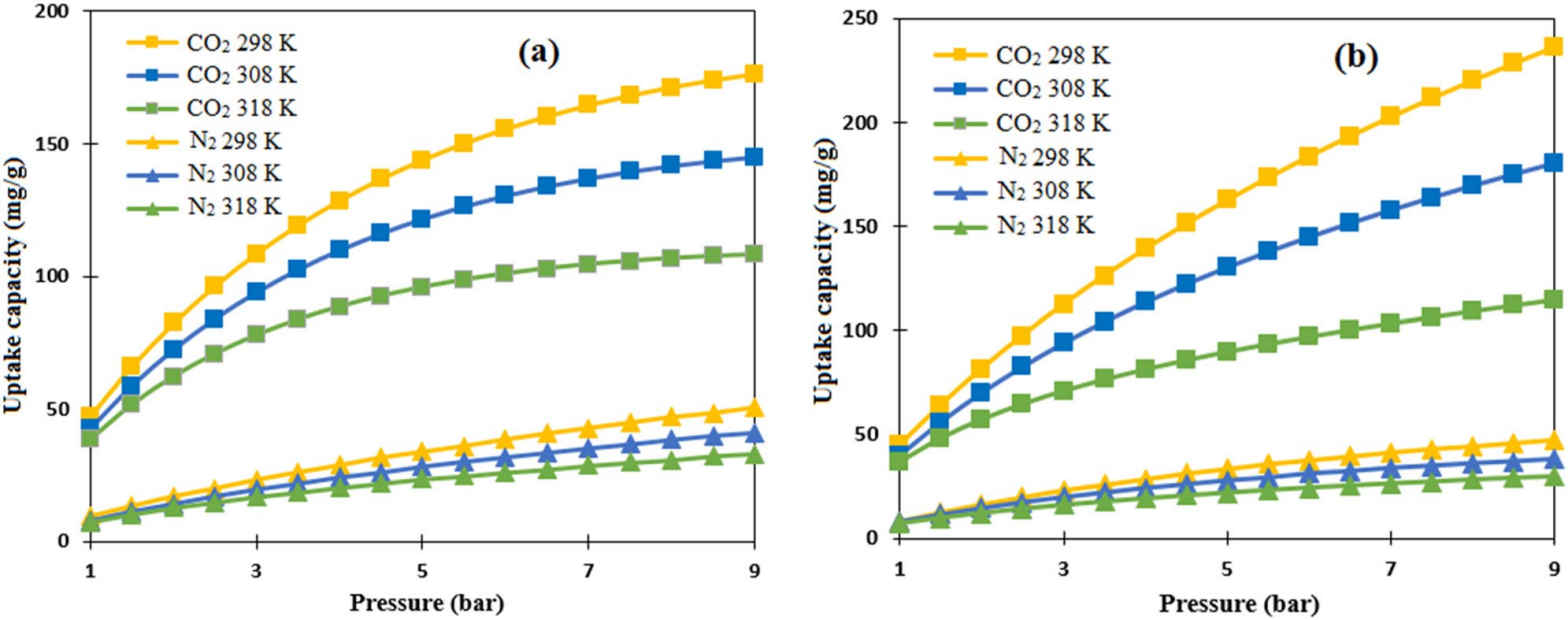
Selective adsorption behavior at CO_2_/N_2_ composition of 15:85 by (**a**) HCP, (**b**) amine functionalized HCP.

Beside the molecular properties of the adsorbate, the operational condition can deeply affected the selectivity of the CO_2_ over N_2_. Therefore, considering the adsorption process pressure and temperature can provide some useful insights about the dependency of the CO_2_/N_2_ selectivity on operational condition. By reviewing the literature, it can be noticed that in industrial gas separation applications, flue gases composition for CO_2_:N_2_ gases rarely exceeds 15:85 (v/v)^72^. So, to investigate the HCP and the amine modified HCP adsorption selectivity by the IAST approach, the gas composition was considered as 15:85 for CO_2_:N_2_ and Langmuir isotherm parameters were used for calculation. The results of the IAST calculation for both type of adsorbents at temperatures of 298 K, 308 K, and 318 K were plotted and shown in Fig. 14. According to this figure, the amine modified HCP sample shows more selective behavior for CO_2_ adsorption than HCP sample in a similar condition, it can be related to enhancing surface electrical properties including dipole-quadrupole moment or polarizability after amine incorporation into HCP structure^73^.

### Comparison between present work and similar studies

A comparative study between this study and similar works on the CO_2_ adsorption by using HCP samples or amine modified HCP samples, was done in this section. The findings of the some similar studies are summarized in Table 6. As reported in this table, the HCP and the aminated HCP samples exhibit high adsorption capacities of q = 301.67 mg/g and q = 414.41 mg/g, respectively. Comparison between this work and other studies result in high peformance and high adsorption capability of the resulting HCP samples for CO_2_ capture applications.

**Table 6:** Some studies on CO_2_ capture by hypercrosslinked polymers and amine-modified polymers.

Adsorbent	Modification agent	Amine loading (wt %)	Operational condition				q (mg/g)	Ref
			T (K)	P (bar)	CO_2_ (%)	Flow rate (ml/min)		
HCP Poly styrene (XAD-4 pc)	Branched-PEI	10, 20, 30, 40, 50	298	1	10	30	62, 96, 142.5, 121.4, 99	^28^
HCP divinyl benzene(PDVB-pc)	–	–	298	1	10	30	24.5	^7^
	DETA	30	298	1	10	30	49.5	
	TETA	30	298	1	10	30	99	
	TEPA	10, 20, 30, 40, 50	298	1	10	30	45, 110, 136.8, 81.4, 28	
1,4-Bis(chloromethyl)benzene	PEI	0, 7.5, 15, 25	298	1	Pure	–	66, 77.6, 81.7, 64.3	^33^
Benzyle chloride base HCP	EDA	0, 64 ( water as solvent), 64 (methanol as solvent)	298	1	Pure	–	75.14, 39.15, 19.10	^29^
CBAP-1 (1–3-5 triphenyl benzene + terephetaloyl chlorid base HCP)	–	–	273, 293, 303	1	Pure	–	140.9, 103.6, 89.8	^30^ ^32^
	EDA	64	273, 293, 303	1	Pure	–	131.9, 97.1, 87.1	
CBAP-2 (biphenyl + 1,3,5-benzenetricarbonyl trichloride base HCP)	–	–-	273, 293, 303	1	Pure	–	141.9, 104.6, 89.1	
	EDA	64	273, 293, 303	1	Pure	–	124, 90.6, 79.0	
PMMA	TEPA	10, 30, 35, 40, 45	308	0.1	10% CO_2_,! 90% He	–	21, 176, 107, 26, 20	
PMMA	PEI	0, 10, 20, 30, 40, 45, 50, 55, 60	348	1	Pure	–	13.6, 26.4, 57.2, 98, 136.5, 149.5, 159.6, 187.44, 66	^27^
HCP-1 (benzene + oxalyl chlorid)	–	–	273	1	Pure	–	22	^31^
	EDA	64					19	
HCP-2 (diphenyl + oxalyl chlorid)	–	–	273	1			68	
	EDA	64					62	
HCP-3 (1,3,5-benzene tricarbonyl + oxalyl chlorid)	–	–	273	1			198	
	EDA	64					175	
HCP-4 (triphenylamine + oxalyl chlorid)	–	–	273	1			165	
	EDA	64					146	
Benzene- HCP	–	–	298	9	pure	–	301.67	This work
Amine functionalized HCP	Primary amine	3.48					414.41	

### Adsorbent regeneration performance

From the economical point of view, the adsorbent reusability is the most important factor for industrial applicability. To investigate the adsorbents recyclability, ten adsorption cycles were conducted at 298 K and 5 bar by both types of adsorbents and the adsorbents were recycled in a vacuum oven at 410 K for 8 h. The amine modified HCP adsorption potential decreased by 3%, and HCP adsorption potential reduced by about 2% after ten cycles. According to the findings, both types of adsorbents can be applicable in industrial applications as high-value adsorbents.

## Conclusion

In this research, the hyper crosslinked polymeric adsorbent from benzene precursor was prepared. To enhance the resulting HCP sample surface’s electrical properties such as dipole moment or polarizability, a chemical modification was done via amine group grafting into the HCP network. In summary, the results of the FTIR and XPS analysis prove successfully grafting of the amine group to the HCP sample skeleton regarding increasing the nitrogen content from 0 to 5.17% after amine modification. The BET analysis results refer to decreasing the specific surface area of the HCP sample from 806 to 453 (m^2^ g^−1^) after surface modification, meanwhile CO_2_ adsorption experiments indicated that amine grafting of the HCP sample increased CO_2_ uptake capacity from 301.67 to 414.41 (mg g^−1^) . Therefore, it can be concluded that a solid sorbent with polar surface and narrow mesopores or micropores can be more suitable for CO_2_ adsorption applications. The findings of the isotherm modeling indicate more appropriating of the Freundlich model, leading multi-layer adsorption of the CO_2_/N_2_ molecules by both types of samples, also the kinetic modeling of the adsorption process refer to the most fitting ability of the fractional order model. The CO_2_/N_2_ adsorption thermodynamic investigation prove the spontaneously and exothermic nature of the CO_2_/N_2_ adsorption by both types of the samples. Comparison of the adsorption selectivity between the HCP sample and the amine grafted HCP adsorbent exhibits higher selectivity for the adsorption of CO_2_ over N_2_ in a specific CO_2_/N_2_ composition of 15:85. The recyclability investigation exhibit minor losses in adsorption efficiency of the adsorbents which reflect the applicability of samples as a high-value adsorbents for industrial applications.

## Data Availability

The datasets used and analyzed during the current study are available from the corresponding author upon reasonable request.

## List of symbols

A: Temkin model constant (L/mol)
b_T_: Temkin isotherm constant (J/mol)
C_e_: Equilibrium concentration
f: Subscript that refers to final condition
i: Subscript that refers to initial condition
k_L_: Langmuir model constant (bar^−^^1^)
k_1_: First order kinetic model rate constant (min^−^^1^)
k_F_: Freundlich model constant (mmol g^−1^ bar^−1/n^)
k_2_: Second order kinetic model rate constant (g mmol^−1^ min^−1^)
k_n_: Fractional-order rate constant for adsorption [(mmol/g)^(1−n)^ min^−m^]
m: Mass of adsorbed gas(mg)
M_w_: Gas molecular weight (g/mol)
P: Pressure (bar)
P^0^: Saturated vapor pressure (bar)
P_e_: Equilibrium pressure (bar)
P^*^: Spreading pressure
x: Mole fraction in adsorbed phase
y: Mole fraction in gas phase
q: Adsorption capacity (mg/g)
q_e_: Equilibrium adsorption capacity (mmol/g)
q_m_: Maximum adsorption capacity (mmol/g)
q_t_: Adsorption capacity at time t (mmol/g)
R: Gas constant (8.314 J K^−1^ mol^−1^)
R^2^: Correlation coefficient
T: Temperature (K)
t: Time (s)
w: Mass of adsorbent (g)
Z: Compressibility factor
ΔH: Enthalpy change
ΔS: Entropy change
ΔG: Gibbs free energy change
α: Elovich model parameter (mmol g^−1^ min^−1^)
β: Elovich model parameter(g mmol^−^^1^)
λ: D–R model constant(mol^2^ J^−^^2^)
ω: Polanyi potential (KJ mol^−^^1^)
EDA: Ethylene diamine
PEI: Poly ethyleneimine
TEPA: Tetra ethylene pentamine
TETA: Tetra ethylene triamine
DETA: Diethylene triamine
HCP: Hyper cross-linked polymer
MPV: Micro pore volume
MPA: Micro pore area
APW: Average pore width
FTIR: Fourier transform infrared
PSD: Pore size distribution
BET: Brunauer Emmett teller
EDS: Energy dispersive X-ray spectroscopy
XPS: X-ray photoelectron spectroscopy
